# The Effect of Mental Fatigue and Gender on Working Memory Performance during Repeated Practice by Young and Older Adults

**DOI:** 10.1155/2021/6612805

**Published:** 2021-10-04

**Authors:** Valentina Pergher, Nele Vanbilsen, Marc Van Hulle

**Affiliations:** ^1^Department of Cognitive Neuropsychology, Harvard University, Cambridge, MA, USA; ^2^Department of Neurosciences, Laboratory for Neuro- & Psychophysiology, KU Leuven-University of Leuven, Belgium

## Abstract

Working memory (WM) is one of the most investigated cognitive functions albeit the extent to which individual characteristics impact on performance is still unclear, especially when older adults are involved. The present study considers repeated practice of a visual *N*-Back task with three difficulty levels (1-, 2-, and 3-Back) in healthy young and older individuals. Our results reveal that, for both age groups, the expected mental fatigue was countered by a learning effect, in terms of accuracies and reaction times, which turned out to benefit females more than males, for all three *N*-Back levels. We conclude that future WM studies, in particular when relying on repeated *N*-Back sessions, should account for learning effects in relation to mental fatigue and gender, in both young and older adults.

## 1. Introduction

Working memory (WM), one of the most investigated cognitive functions, refers to the ability to temporary store and manipulate information necessary to complete complex tasks for a short period of time [1, 2]. Literature suggests that WM is the outcome of a series of processes that manage two domain-specific slave systems [3]. One subdomain is the phonological store that elaborates verbal information; the other is the visual cache that elaborates visuospatial information [3].

Previous studies have indicated that individual differences, such as age, gender, and education, may have a substantial impact on several cognitive domains [4]. In the results after a single session of cognitive performance, the impact of age was observed by lower accuracies and increased reaction times for older individuals compared to younger ones [5–7]. Some studies found lower accuracies based on task demand [8], while in the study of Hale et al. [9], age-related performance decline was independent from task demand. In general, it has been shown that WM performance becomes worse with aging, indicating an increased reaction time due to a general slowdown in information processing [10, 11] or due to a higher difficulty in suppressing interference from irrelevant information [12]. Moreover, education level has been shown to provide a protective effect on age-related changes during a verbal learning task [13–15]. Meijer et al. [13] showed that only participants with a high educational level were able to inhibit irrelevant verbal information. Additionally, a higher education level indicated to play a significant positive role when individuals aged [16].

Albeit there is a broad consensus on the impact of age and education level on cognitive functioning, this is less in the case with gender. For instance, there are stark discrepancies between studies as several WM tasks failed to reveal any gender differences [17], whereas others observed significant age-related changes in task performance [18] and neurofunctional responses [19]. Several studies that focused on adolescents [20], young adults [21, 22], and older adults [23] did not observe any significant gender differences. In contrast, other studies demonstrated a gender difference for WM tasks by showing that women perform better than men, especially in spatial-based tasks [24, 25], although the picture could change when considering higher WM loads [26]. Furthermore, a recent meta-analysis that investigated gender differences in WM showed a female advantage for object location memory and a male advantage for *N*-Back tasks [27]. Several studies reported a superior female performance for object-location memory tasks [28–30] and for verbal tasks [31, 32]. However, not all high-load WM studies reported a gender-related difference. For instance, Goldstein et al. [33] observed no effects for gender when using a task similar to the letter *N*-Back task in a behavioral fMRI study, although female subjects had a higher neural activation in the prefrontal cortex. Despite the evidence, gender has been much less researched in the context of the visual (object) *N*-Back task, especially in older individuals, and the few studies that have been conducted showed mixed outcomes. The study of Speck et al. [34] showed a female advantage for a verbal *N*-Back task that uses sequences of displayed words and numbers. Using a digit *N*-Back task, Pliatsikas et al. [18] showed that, with increasing age, males exhibited a steeper WM decline than females and that, with increasing education levels, females reached greater WM gains than males. However, the study of Lejbak et al. [28] revealed a male advantage when using an object-location memory task (*N*-Back), and Schmidt et al. [17], using a verbal *N*-Back task in young adults, demonstrated that WM is not affected by gender at behavioral and neural levels.

Considering that males and females have different pathways to encode memories, solve problems, and make decisions, these gender-related functional differences may be associated with gender-specific structures of the brain [35]. Xin et al. [36] showed, using a 3D “Pure” Convolutional Neural Network (PCNN), brain structure differences between males and females. Specifically, they found 25 brain features that were significantly different between men and women. These structures included the left precuneus, left postcentral gyrus, left cingulate gyrus, right orbital gyrus of the frontal lobe, left occipital thalamus in the gray matter volume, middle cerebellum peduncle, genu of corpus callosum, right anterior corona radiata, right superior corona radiata, and left anterior limb of the internal capsule. Therefore, Xin et al. [36] confirm that gender-related differences in the brain structure exist and that they might explain gender-related differences in cognition. Furthermore, another potential source of gender differences in WM task performance is the gender-specific nature of the adopted strategy as shown by Alarcón et al. [20]. In this study, using a spatial WM task in adolescents, no gender differences in performance were found, although significant differences in patterns of neural activity measured by fMRI were detected. Females showed a reduced activation of the default mode network, while males an activation in regions associated with spatial WM function, suggesting that there might be multiple networks associated to WM and that changes in information-type such as spatial WM tasks might reveal the adoption of gender-specific behavioral strategies [37]. Furthermore, females showed an advantage relative to males on tests that measure recollection, processing speed, object location memory, verbal memory, verbal learning, and precision tasks [38, 39]. In contrast, other studies showed a male advantage in tests that require mental rotation or manipulation of an object [40] and in reaction time and finger tapping [38]. Moreover, Upadhayay and Guragain [41] investigated the effect of progesterone on cognitive functions when comparing several cognitive task performances between males (one time) and females (two times: during preovulatory and postovulatory phases of the menstrual cycle). While studies have shown that estrogen and testosterone accentuate cognitive functions, the role of progesterone on cognitive functions is still controversial. The results indicated that male cognitive functions were comparable to female preovulatory phase cognitive functions. However, females, during the postovulatory phase of their cycle, may have advantages in executive tasks (Stroop test), but disadvantages in attentional tasks.

One of the most common WM paradigms used to study complex cognitive functions is *N*-Back, a task that requires deciding whether the current stimulus matches the one presenting N stimuli before. It calls upon different mental processes [42], in particular WM updating that involves the encoding of incoming stimuli, monitoring, maintenance and updating of the sequence, and matching the current stimulus to the one presenting N positions back. Furthermore, the *N*-Back task also reflects other high-order cognitive functions, such as inhibitory control, cognitive flexibility, problem solving, and decision making [2]. Recent functional imaging studies focused on the *N*-Back task and investigated structural correlates for different difficulty levels. Indeed, the *N*-Back task requires a continuous variable load and, consequently, different cognitive efforts [43]. For instance, using a visual *N*-Back task, Pesonen et al. [44] observed differences in alpha band power when participants were performing a higher *N*-back level (2- and 3-Back) compared to a lower one (1-Back), confirming that information maintenance and manipulation load change with increasing N [45]. Moreover, it has been shown that with age, cognitive performance becomes slower and less accurate, and performance accuracy in more complex WM tasks decreases [46]. Another factor of interest, albeit only rarely investigated, is mental fatigue resulting from prolonged periods of cognitive activity. It is associated with tiredness or exhaustion resulting in a decrease in task performance and commitment [47]. Käthner et al. [48] and Kohlmorgen et al. [49] highlighted that, along with a lower performance, alpha band power increased with mental fatigue in older individuals, respectively, during dichotic listening tasks and in real operating environments for the last run compared to the first run. Furthermore, also, the study of Pergher et al. [50] attributed an important role to mental fatigue, comparing the first and last run of an object *N*-Back task, and revealed significant differences in alpha band power of older adults.

In the present study, we investigated accuracy and reaction time during repeated sessions (lasting less than one hour) of an *N*-Back task with three difficulty levels by healthy young and older adults. Both age groups performed the 1-, 2-, and 3-Back levels, for a total of four rounds each. We hypothesize that the last part of the experiment (last round) compared to the first part (first round) has a higher accuracy level due to a learning effect. Indeed, it has been shown that repetition of visual stimuli in certain cognitive tasks can promote improvements in implicit memory and attention [51]. However, based on the studies of Käthner et al. [48] and Kohlmorgen et al. [49], demonstrating the effect of mental fatigue, especially in older individuals, we investigated the effect of the latter performing our analyses on the first (1^st^) and last (4^th^) rounds of the *N*-Back task. We expected that the effect of mental fatigue was stronger compared to the learning effect, especially in older adults, showing a lower accuracy at the end of the 1-hour *N*-Back task performance. Besides the possible impact of fatigue, we are aware that learning could also interfere with task performance. Moreover, taking into consideration the controversial results of gender on the WM domain [23, 24], especially in the elderly population, we also explored the impact of gender on an object *N*-Back task. Based on previous literature on *N*-Back studies [18], we hypothesize an advantage for females compared to males.

## 2. Materials and Methods

### 2.1. Participants

We recruited 23 young healthy subjects (14 females, mean age 24.78, age range 19–34 years) via flyers and advertisements, all undergraduate or graduate students from KU Leuven University, and 16 older healthy subjects (8 females, mean age 62.12 years, range 52–69 years). Demographics are shown in Table 1.

Participants were included in the study only if they met the following inclusion criteria: no history of psychiatric or neurological disease, normal or corrected vision, and not on any medication that could interfere with cognitive functioning. Participants were divided according to age and gender as follows: female (*N* = 14, education = 14.86 y ± 2.88) and male (*N* = 9, education = 12.88 y ± 2.52) for young adults; female (*N* = 8, education = 7.25 y ± 1.77) and male (*N* = 8, education = 7.62 y ± 2.13) for older adults. All participants, except four, were part of previous studies on the effect of WM training on behavioral and EEG responses ([52] for older adults; [53] for young adults). Note that due to some technical issues when recording behavioral responses of 4 young participants, we retained 19 subjects in total for reaction time (RT) outcomes of which 10 females and 9 males.

### 2.2. *N*-Back Task

**Figure 1:**
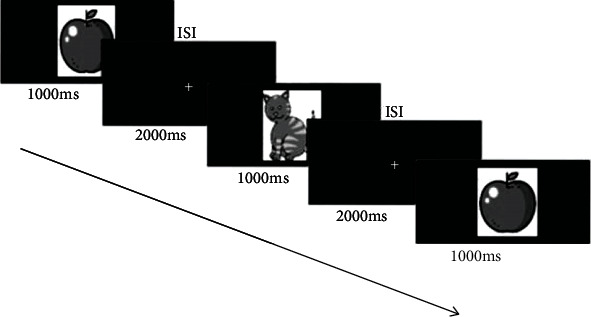
Example stimulus sequence during 2-Back task with stimulus durations of 1000 ms and an interstimulus interval (ISI) of 1500 ms.

We used an object *N*-Back task as in our previous studies [50, 52]. The task requires to decide whether the current stimulus matches the one presenting *N* items before (Figure 1). We divided the task into three difficulty levels, 1-Back, 2-Back, and 3-Back, respectively. A given *N*-Back level consisted of four blocks (or rounds), and each block contains 100 stimuli. For each block of 100 stimuli, 33% of them were targets. The stimuli were presented for 1 s followed by a 1.5 s interstimulus interval (ISI) and 0.5 s of feedback presentation (red frowny/green smiley). Between blocks, the subject could take a break and continue when pressing the space bar. Subjects performed the *N*-Back task within 60 minutes.

### 2.3. Procedure

Prior to the experiment, participants were informed about the experimental procedure and signed an informed consent form. Participants filled out a brief demographic questionnaire. All participants were invited to the laboratory for a single session of the *N*-Back task with all three task difficulty levels, as described above, repeated four times (further called “rounds”). The study was approved by our university hospital's ethical committee (UZ KU Leuven, S59475). Participants received a payment of 20 euros.

### 2.4. Statistical Analyses

All analyses were performed using Matlab (version 2019b). The *N*-Back task results were analyzed using a two-way ANOVA with accuracy or reaction time (RT) as dependent variable and *N*-Back level and gender as independent variables. The Shapiro-Wilk test [54] was applied to assess whether normality was violated, considering our relatively small sample size. If normality was violated, a more conservative *p* value of 0.01 was adopted. Post hoc analyses were calculated using independent samples *t*-tests with *N*-Back level or gender as independent variable and accuracy or RT as dependent variable, and correction for multiple comparison (Bonferroni) was used. Furthermore, we separated the first and last round *N*-Back performance, considering as first round the first 100 stimuli and as last round the last 100 stimuli of the same *N*-Back level. In order to address the possible effect of imbalanced gender, we ran a bootstrapping procedure (using bootstrap sampling, accessible via Matlab Central), for which we created two equally sized groups (*N* = 8/*N* = 8) of male and female accuracies (in % correct responses) by sampling the corresponding (with replacement) original *N* = 14/*N* = 9 male and female accuracies, and determined the *p* value of the difference in accuracy and its confidence interval (CI). We repeated this procedure 10000 times and used the obtained difference distribution to estimate the *p* value of the actual difference in mean.

Finally, *η*p2 = 0.098 [55] were reported (*η*^2^ = SSeffect/SSeffect + SSerror, where SS is sum of squares) to indicate the magnitude of the significant differences for behavioral outcomes.

## 3. Results

### 3.1. Mental Fatigue in Reaction Time and Accuracy

**Figure 2:**
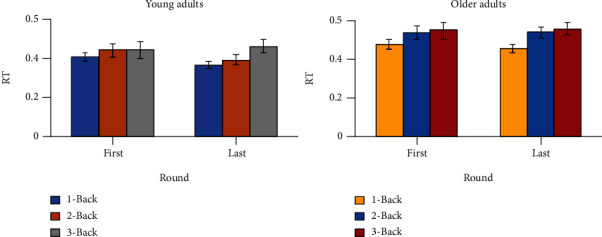
Means ± SD for reaction time (RT) for young (a) and older (b) adults comparing first and last round during *N*-Back task performance for the three different *N*-Back levels.

We compared reaction times (RTs) of the first and last rounds of the *N*-Back task to detect a possible effect of mental fatigue (Figure 2). We did not find any significant difference in RT when comparing the first and last rounds in younger adults. When looking at older adults, our results showed a significant main effect for *N*-Back level (*F*(2, 90) = 4.89, *p* = 0.010, *η*p2 = 0.098), indicating that, overall, older adults exhibited lower RTs for 1-Back compared to 2- and 3-Back. However, no significant differences across levels were found when comparing the first and last rounds.

**Figure 3:**
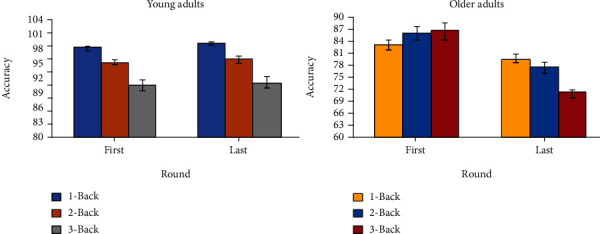
Means ± SD for accuracy for young (a) and older (b) adults comparing first and last round during *N*-Back task performance for the three different *N*-Back levels.

Similarly, we compared the accuracy of the first and last rounds of the *N*-Back task (Figure 3). Our results showed a significant difference for *N*-Back level in younger adults (*F*(2,126) = 71.76, *p* < 0.001, *η*p2 = 0.533) but not for round; for older adults, our results showed a significant difference for both round (*F*(1, 60) = 7.35, *p* = 0.009, *η*p2 = 0.109) and *N*-Back level (*F*(2, 60) = 15.24, *p* < 0.001, *η*p2 = 0.337).

### 3.2. Gender Differences in Reaction Time

**Figure 4:**
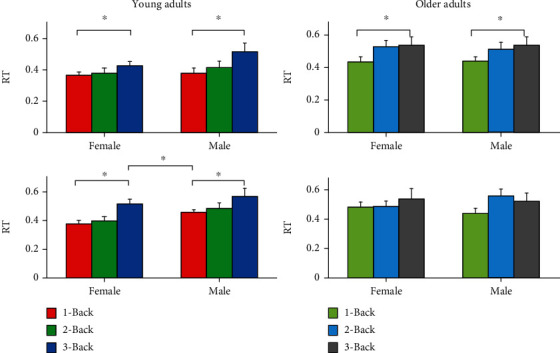
Means ± SD for reaction time (RT) for young (a) and older (b) adults comparing females and males during *N*-Back task performance for the three different *N*-Back levels.

To detect further differences, we used a two-way ANOVA (gender × *N* − Back level) for the first and last round separately (Figure 4). Our results showed significant differences for young adults in the first round for gender (*F*(1, 51) = 5.50, *p* = 0.023, *η*p2 = 0.097) and *N*-Back level (*F*(2, 51) = 8.34, *p* = 0.001, *η*p2 = 0.246). Looking at the fourth round, our results showed a significant main effect for *N*-Back level (*F*(2, 51) = 4.13, *p* = 0.022, *η*p2 = 0.139).

We performed the same analyses for older adults and found significant differences only in the fourth round for *N*-Back level (*F*(2, 42) = 3.94, *p* = 0.027, *η*p2 = 0.158). For both rounds, no significant differences between males and females were found.

Our post hoc analyses of gender revealed a significantly shorter RT for young females (*M* = 0.431; SD = 0.019) compared to young males (*M* = 0.497; SD = 0.025) in the first round (*t*(55) = 2.1101, *p* = 0.0394), considering all three *N*-Back levels.

Furthermore, our post hoc analyses of *N*-Back level showed significant differences in RT for young adults in the first round between 1-Back (*M* = 0.406; SD = 0.021) and 3-Back (*M* = 0.542; SD = 0.030) (*t*(36) = 3.6426, *p* < 0.001) and between 2-Back (*M* = 0.440; SD = 0.022) and 3-Back (*M* = 0.542; SD = 0.030) (*t*(36) = 2.7030, *p* = 0.0104) and in the fourth round between 1-Back (*M* = 0.036; SD = 0.017) and 3-Back (*M* = 0.459; SD = 0.031) (*t*(36) = 2.6634, *p* = 0.0115). Similarly, our results showed significant RT differences for older adults between 1-Back (*M* = 0.461; SD = 0.016) and 2-Back (*M* = 0.535; SD = 0.021) (*t*(62) = 2.8631, *p* = 0.0057) and between 1-Back (*M* = 0.461; SD = 0.016) and 3-Back (*M* = 0.549; SD = 0.026) (*t*(62) = 2.9162, *p* = 0.0049) when taking the first and last rounds together. Furthermore, we found significant differences in RT of older adults in the fourth round between 1-Back (*M* = 0.448; SD = 0.020) and 2-Back (*M* = 0.534; SD = 0.027) (*t*(30) = 2.5631, *p* = 0.0156) and between 1-Back (*M* = 0.448; SD = 0.020) and 3-Back (*M* = 0.5531; SD = 0.033) (*t*(30) = 2.7171, *p* = 0.0108).

### 3.3. Gender Differences in Accuracy

To detect significant accuracy differences during the first and last round, we used a two-way ANOVA (gender × *N* − Back level). Our results showed significant differences in the first round for young adults for gender (*F*(1, 60) = 7.92, *p* = 0.007, *η*p2 = 0.117) and *N*-Back level (*F*(2, 60) = 50.86, *p* < 0.001, *η*p2 = 0.629) and in the fourth round for gender (*F*(1, 63) = 27.45, *p* < 0.001, *η*p2 = 0.306), *N*-Back level (*F*(2, 63) = 51.45, *p* < 0.001, *η*p2 = 0.620), and gender × *N* − Back level (*F*(2, 63) = 4.92, *p* = 0.010, *η*p2 = 0.135). Furthermore, we performed the same analyses for older adults and found significant differences in the fourth round for *N*-Back level (*F*(2, 42) = 3.46, *p* = 0.041, *η*p2 = 0.141), but not in the first round. No significant differences for gender across *N*-Back levels were found in both rounds.

Our post hoc analyses of young adults for gender revealed a trend for higher accuracies for females (*M* = 95.833; SD = 0.621) compared to males (*M* = 93.796; SD = 0.0986) (*t*(64) = 1.837, *p* = 0.0707) in the first round, considering all three *N*-Back levels.

**Figure 5:**
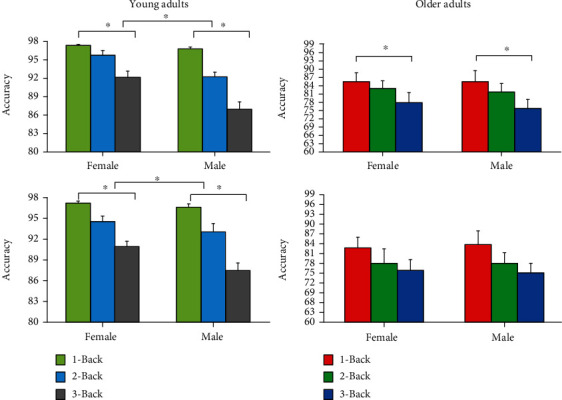
Means ± SD for accuracy for young (a) and older (b) adults comparing females and males during *N*-Back task performance for the three *N*-Back levels.

Moreover, our post hoc analyses of young adults for *N*-Back level showed significant differences in accuracy, when considering the first and last rounds jointly, for 1-Back (*M* = 99.130; SD = 0.168) and 2-Back (*M* = 95.853; SD = 0.513) performance (*t*(86) = 6.2951, *p* < 0.001), between 1-Back (*M* = 99.130; SD = 0.168) and 3-Back (*M* = 91.023; SD = 0.657) performance (*t*(88) = 12.1853, *p* < 0.001), and between 2-Back (*M* = 95.853; SD = 0.513) and 3-Back (*M* = 91.023; SD = 0.657) performance (*t*(84) = 5.7557, *p* < 0.001) for young adults (Figure 5). Furthermore, our results showed significant differences in older adults between 1-Back (*M* = 79.091; SD = 0.012) and 2-Back (*M* = 75.606; SD = 1.220) performance (*t*(42) = 2.1981, *p* = 0.0335), between 1-Back (*M* = 79.091; SD = 0.012) and 3-Back (*M* = 71.477; SD = 0.824) performance (*t*(42) = 5.8324, *p* < 0.001), and between 2-Back (*M* = 75.606; SD = 1.220) and 3-Back (*M* = 71.477; SD = 0.824) performance (*t*(42) = 2.8042, *p* = 0.0076), also when considering the first and last rounds jointly. Moreover, significant differences between rounds were found in older adults. Specifically, accuracy level was higher for the last round (*M* = 76.919; SD = 1.042) compared to the first round (*M* = 73.864; SD = 0.878) (*t*(64) = 2.2427, *p* = 0.0284).

Furthermore, we found significant differences for young adults in the fourth round between 1-Back (*M* = 99.456; SD = 0.186) and 2-Back (*M* = 96.341; SD = 0.684) performance (*t*(44) = 4.3923, *p* = 0.0143), between 1-Back (*M* = 99.456; SD = 0.186) and 3-Back (*M* = 91.594; SD = 1.008) performance (*t*(44) = 7.6730, *p* < 0.001), and between 2-Back (*M* = 96.341; SD = 0.684) and 3-Back (*M* = 91.594; *SD* = 1.008) performance (*t*(44) = 3.8962, *p* < 0.001). Also, we found significant differences in the first round between 1-Back (*M* = 98.804; SD = 0.266) and 2-Back (*M* = 95.476; SD = 0.713) performance (*t*(44) = 4.3923, *p* = 0.0143), between 1-Back (*M* = 98.804; SD = 0.266) and 3-Back (*M* = 90.568; SD = 0.808) performance (*t*(43) = 9.8538, *p* < 0.001), and between 2-Back (*M* = 95.476; SD = 0.713) and 3-Back (*M* = 90.568; SD = 0.808) performance (*t*(41) = 4.5382, *p* < 0.001). Similarly, we found significant differences for older adults in the fourth round between 1-Back (*M* = 86.198; SD = 2.417) and 2-Back (*M* = 82.917; SD = 2.160) performance (*t*(42) = 4.5272, *p* < 0.001).

In order to address the possible effect of imbalanced gender, we ran a bootstrapping procedure (see Materials and Method and Statistical Analyses, for further details). We obtained a *p* value of 0.0273, with a 95% CI in mean accuracy difference of [0. 178572, 1.369047], for young adults in the last (fourth) round for 1-Back, indicating a significant higher accuracy for females compared to males. Moreover, we ran the bootstrapping test which returned a *p* value of 0.0009, with a 95% CI of [1.574074, 6.296297], for young adults in the last round for 2-Back, indicating a significant higher accuracy for females compared to males. Last, we ran the bootstrapping test that showed a *p* value of 0.0010, with a 95% CI of [2.407407, 9.259259], for young adults in the last round for 3-Back, indicating a significant higher accuracy for females compared to males. Furthermore, we applied the same method for the first round in young adults. The bootstrapping test returned a *p* value of 0.166, with a 95% CI of [-0.462963, 1.851852], for 1-Back, indicating a nonsignificant accuracy difference between females and males. Also, the bootstrapping test showed a *p* value of 0.1376, with a 95% CI of [-1.296297, 4.537037], for 2-Back, indicating a nonsignificant accuracy difference between females and males. Last, the bootstrapping approach returned a *p* value of 0.0097, with a 95% CI of [0.648148, 6.574074], for 3-Back, indicating a significant higher accuracy for females compared to males. To summarize, our results showed that females performed better than males in the last round for all three *N*-Back levels and in the first round for the 3-Back task. No significant differences were found in the first round for the 2 and 3-Back levels between females and males.

.

## 4. Discussion

We investigated the accuracy and reaction time of a demanding WM task, called *N*-Back, for three different difficulty levels, in healthy young and older adults. Based on the studies of Käthner et al. [48] and Kohlmorgen et al. [49], which demonstrated the impact of mental fatigue between the beginning and the end of cognitive performance, especially in older individuals, we split our *N*-Back data into first (1^st^) and last (4^th^) rounds. However, besides mental fatigue, learning might be a moderator of improved performance outcomes [51]. The *N*-Back task has been commonly shown to depend on individual characteristics such as age and education level. However, the influence of gender has largely been neglected, especially in older individuals, and the few studies that have been conducted returned inconsistent results [17, 19]. Speck et al. [34] observed a female advantage for a verbal *N*-Back task that uses sequences of displayed words and numbers and reported gender-specific neural patterns. On the other hand, the study of Lejbak et al. [28] revealed a male advantage when using an object-location memory task (*N*-Back) in young adults. Schmidt et al. [17], using a verbal *N*-Back task in young adults, observed that WM is not affected by gender at behavioral and neural levels, while Pliatsikas et al. [18], using a digit *N*-Back task in older adults, showed that with increasing age, males exhibited a steeper WM decline than females, and with increasing education, females exhibited greater WM gains than males. Similarly, Lejbak et al. [24] reported a gender-related difference in favor of females for an object location memory task, in young adults. McCarrey et al. [23] showed that males outperformed females in two visuospatial ability tasks and that females outperformed males in most other tests of cognition. However, gender differences over time revealed steeper rates of age-related decline for men on measures of mental status, perceptuomotor speed and integration, and visuospatial ability. Taking into consideration these studies, we hypothesized an advantage for females compared to males.

Our results showed that mental fatigue did not affect *N*-Back task performance in both young and older adults, but instead, we observed a higher accuracy level in the last round compared to the first round for older adults, suggesting that mental fatigue was countered by a learning effect. Indeed, although mental fatigue might be present during the last part of cognitive task performance, learning is another factor that could interfere with performance. It has been shown that repeated practice of a cognitive task involving visual stimuli can improve implicit memory and attention processes [51, 56], resulting in increased performance even within one hour. This process has been defined as fast perceptual learning (PL) [51, 57]. Beside this, a persistent higher accuracy level was observed for the easier 1-Back level compared to the more difficult 2- and 3-Back levels.

Furthermore, according to our expectations regarding gender, the outcomes of young subjects were in line with the results observed by Pliatsikas et al. [18] and Speck et al. [34], as we found a general female advantage for both accuracy and RT for the *N*-Back task performance. Additionally, our bootstrapping test, which we used to balance the number of females and males in the group of young adults, confirmed that females enjoy higher accuracy levels compared to males in all three *N*-Back levels, especially in the last round. Further studies should focus on the underlying factors producing these differences, e.g., whether the stimuli used were more or less familiar. One hypothesis is that the male advantage can be explained by a better performance in terms of semantic memory for common objects [28], whereas worse male performance has been seen for other object categories, such as words, numbers, shapes, faces, and less familiar objects. Another possibility can be attributed to gender-related differences in brain structure as demonstrated by Xin et al. [36] which might be reflected by gender-related differences in cognition. An additional hypothesized moderator could be the specific strategy used to perform the task instead of the task per se [58]. From this perspective, tasks using different stimulus types could not be the prime reason for differences in outcome, whereas the cognitive processes used to solve the WM task may instead play an important role in accuracy and RT responses [37]. Moreover, Upadhayay and Guragain [41] suggested that hormones, especially progesterone, could have a significant impact on cognitive performance. While studies have already shown that estrogen and testosterone accentuate cognitive functions, their study indicated that male cognitive functions were comparable to female preovulatory phase cognitive functions, although females, during the postovulatory phase of their cycle, enjoyed advantages in executive tasks and disadvantages in attentional tasks, compared to males.

The present study has important implications in terms of behavioral outcomes. We examined whether accuracy and RT additional affected WM task performance as well as mental fatigue and learning. Importantly, we showed that gender plays an important role in young, but not in older adults. However, previous studies indicated that gender does not contribute to differences in behavioral outcomes [23], while in contrast, other studies [33, 34] reported differences in neural activation pathways of males and females. Although the *N*-Back task has been commonly used for assessing WM in a variety of populations, such as young and older subjects, and individuals with neurological disorders, the role of gender in cognitive performance in older adults is still elusive.

Our findings should be considered in light of some limitations. It has been shown that increasing stimulus duration improves memory performance during retention of visuospatial information [50, 59]. Consequently, it is important to note that, similar to the study of Speck et al. [34], our stimuli were presented for 1 second, whereas most of the studies used a longer stimulus duration [60, 61]. This factor might have affected our RT and accuracy outcomes. Moreover, in this study, we used an object *N*-Back task; however, further studies should compare different *N*-Back task modalities (animals, human faces, geometric shapes, and inanimate/animate stimuli), which have been considered in the study of Lejbak et al. [28] albeit they considered only a 2-Back task (hence, no variation in *N*-Back level). Also, our final outcomes showed gender difference only for young, but not older adults. This pattern could reflect a ceiling effect; however, if true, then we probably would have seen some gender differences in at least one of the three *N*-Back difficulty levels, which is not the case. We suggest more research to address this issue. While here we reported accuracy and RT outcomes, combining neuroimaging data recorded when performing an *N*-Back task could be a worthwhile addition to study gender differences. Last, our test sample was limited to examine both the effect of age and gender. A large test sample size should be used to verify whether the effect of gender in young but not in older adults could be replicated. The reason behind the small groups is due to the fact that most of the participants came from our previous studies ([52] for older adults; [53] for young adults) that investigated the effect of WM training, both in terms of behavioral and EEG responses, requiring several in-person training sessions for each subject.

## Figures and Tables

**Table 1:** Participant demographics showing gender division, mean age, and years of education (standard deviation between brackets).

	Young adults (*N* = 23)	Older adults (*N* = 16)
Age	24.78 (3.99)	62.12 (4.69)
Gender	9 M/14 F	8 M/8 F
Education (in years)	14.3 (2.94)	7.5 (1.9)

## Data Availability

The data of this study are available via this link: https://drive.google.com/drive/folders/1eFhk33uOAfdyiHBuf4olwoIUgOxBHdRo?usp=sharing.
